# Implementing learning health systems in the UK NHS: Policy actions to improve collaboration and transparency and support innovation and better use of analytics

**DOI:** 10.1002/lrh2.10209

**Published:** 2019-12-15

**Authors:** Sarah Scobie, Sophie Castle‐Clarke

**Affiliations:** ^1^ Nuffield Trust London UK

**Keywords:** analytics, digital policy, implementation, innovation, learning health system, transparency

## Abstract

Learning health systems (LHS) use digital health and care data to improve care, shorten the timeframe of improvement projects, and ensure these are based on real‐world data. In the United Kingdom, policymakers are depending on digital innovation, driven by better use of data about current health service performance, to enable service transformation and a more sustainable health system.

This paper examines what would be needed to develop LHS in the United Kingdom, considering national policy implications and actions, which local organisations and health systems could take.

The paper draws on a seminar attended by academics, policymakers, and practitioners, a brief literature review, and feedback from policy experts and National Health Service (NHS) stakeholders.

Although there are examples of some aspects of LHS in the UK NHS, it is hard to find examples where there is a continuous cycle of improvement driven by information and where analysis of data and implementing improvements is part of usual ways of working.

The seminar and literature identified a number of barriers. Incentives and capacity to develop LHS are limited, and requires a shift in analytic capacity from regulation and performance, to quality improvement and transformation. The balance in priority given to research compared with implementation also needs to change.

Policy initiatives are underway which address some barriers, including building analytical capacity, developing infrastructure, and data standards. The NHS and research partners are investing in infrastructure which could support LHS, although clinical buy in is needed to bring about improvement or address operational challenges.

We identify a number of opportunities for local NHS organisations and systems to make better use of health data, and for ways that national policy could promote the collaboration and greater use of analytics which underpin the LHS concept.

## 1 INTRODUCTION

The UK National Health Service (NHS) has developed a long‐term plan,^1^ which places digital developments at the heart of steps to improve health and care and deliver services in a sustainable way. The plan recognises that a huge—but currently underdeveloped—benefit of digitising the delivery of care is using the accumulated data about care activities, resources, and outcomes to improve services. Experience from health care and beyond suggests that in order to realise the benefit of adopting new technology requires that data should be used to transform services, rather than simply digitising current ways of working.

Box 1 **initiatives for building analytical capacity in the United Kingdom**

Building the digital ready workforce^24^ programme is composed of a series of workstreams focussing on leadership and culture, professionalisation, the Digital Academy (which provides specialist training to CIOs and CCIOs), and digital literacy for NHS staff. The programme also funds and supports The Faculty of Clinical Informatics,^25^ the professional body for health, and social care professionals working in informatics. It is also working on campaigns to attract people with digital skills to the NHS.

The Skills Framework for the Information Age (SFIA^26^) is a model for describing and managing competencies for information technology professionals for the 21st century, and is intended to help match the skills of the workforce to the needs of the business. It is not specific to the health sector, but provides a framework to articulate the skills and the level of responsibility needed for organisations using digital in the 21st century.

The Association of Professional Healthcare Analysts (APHA)^27^ aims to raise the profile of health care analysts and provide a professional support network, ultimately achieving professional registration status for its members. The intention is to drive up the quality and applicability of robust analytics as an aid to evidence‐based decision making in a modern health and care system. APHA is associated with the Federation of Informatics Professionals.^28^

Box 2 **developing the infrastructure for LHS in the NHS**

Health Data Research UK (HDR UK)^41^ is a joint investment led by the Medical Research Council, working in partnership with academia, NHS, Government, industry, and charities. It aims to harness health and biomedical data for discovery research in the United Kingdom, and develop and apply cutting edge data science approaches in order to address the most pressing health research challenges facing the public.

The Digital Innovation Hub^42^ programme, which is part of HDR UK infrastructure, is developing technology and methods to support the use of large data sets and analytical methods for applied health research, evaluation, and innovation. The aim is to develop between 3 and 5 hubs in regions across the United Kingdom to connect health‐related data for applied research and innovation within a single interoperable, trusted, and secure governance framework. This will enable accredited researchers, scientists, and innovators to work together and safely and securely use data to harness scientific knowledge and emerging technologies at scale across populations of between 3 and 5 million people.

The NHS in England has established the Local Health and Care Record (LHCR^43^) programme, which is intended to build on existing projects and create a set of national standards that all local health and care record initiatives across England will be required to follow.^44^ The first wave of LHCRs are in areas with preexisting shared record programmes, which already have a track record in developing platforms for sharing and accessing data for analysis. It is anticipated that LHCRs will deliver standardised approaches to how data is stored^45^ to enable analysis of data across multiple areas.

Box 3 **data standards in the UK NHS**

Improving data quality at source is critical, but not sufficient for LHSs. Linking data across multiple sources requires the ability to know when two items of the data are the same—either by using common data standards and codes or from having a mapping to translate codes between different sources. The NHS data dictionary provides a strong starting point for this, in contrast to social care, where there are currently minimal data standards.

Many of the existing NHS standard dataset definitions need revision because they are based around care activities and pathways that are changing. For example, care activity that historically has taken place in an outpatient appointment could happen through a virtual or online consultation, through advice was provided directly from a specialist to a GP, and be delivered by a range of different clinical professionals in a range of settings.

Responsibility for the standards for collecting and publishing data sits with NHS Digital,^54^ leadership for standardising records for patient care sits with the Professional Record Standards Body,^55^ but implementation of the standards has not always taken place.

Learning health systems depend on high quality data being captured during the care of the individual patient,^56^ and so improving the implementation of standards for data recording, and aligning health record standards with data standards will support the development of LHS.

The Learning Health System (LHS) concept,^2^ in which data and analytics is part of a continuous cycle with implementation and improvement, is gaining traction in the United Kingdom as an approach to bring evidence into practice much more rapidly, in order to drive improvement.

This paper examines the state of LHS development in the United Kingdom, the context in which analytics is currently undertaken, potential barriers to LHS, and actions that could be taken by national policy makers and by local health systems to accelerate the use of health data for improving health care.

The paper draws on a seminar on LHS held at the Nuffield Trust, an independent health policy think tank, and on evidence and experience from the United Kingdom and internationally.

## 2 THE UK NHS: POLICY CONTEXT

The health system in the United Kingdom^3^ is funded through general taxation, and is largely free at the point of use. The majority of health service provision is by services which are managed by the NHS, although there are some non‐NHS providers, particularly for elective care and some community services. Primary care has a key role in providing universal health services, supporting long‐term conditions, and managing access to secondary services, and is delivered by largely independent general practitioners, working under a national contract.

NHS policy^1^ is increasingly focused on improving integration of services across geographic areas, both to improve coordination of care, and to support population health management. This is envisaged to enable the NHS to deliver services more effectively to the growing number of patients with multiple long‐term conditions.

The NHS has had a chequered history of digital development^4^. A major national programme to deliver system‐wide digitisation during the 2000's was dismantled in 2011: despite some successes, the policy failed to deliver its intended vision. A 2016 report by the National Advisory Group on Health Information Technology in England found that the programme was overly centralised, lacked clinical buy‐in, overlooked the importance of adaptive change, and had a politically‐driven agenda.^5^ More recently,^6^ policy has focused on building capacity and capability for locally tailored solutions, working to national standards.

### 2.1 Learning health systems in the United Kingdom

The LHS^7^ concept has developed over the last decade, as clinicians have identified new opportunities to use electronic health data to improve services as part of the ongoing delivery of care, rather than as separate research activity. Briefly, the learning health system cycle captures current care and outcomes by analysing data from clinical encounters; combines local knowledge with evidence from elsewhere to understand quality of care and how it could be improved; and turns this knowledge into action, which is undertaken by the learning community.

A key feature of LHS, which differentiates them from research, is that for each improvement project there is a learning health community^2^ that takes on the responsibility for acting on the learning, not just creating new evidence. Proponents of LHS have identified key characteristics of learning health communities, which need to be: all inclusive, involving both the whole health care team and the patient, trusted, decentralised, and reciprocal, such that participants creating data also receive access to that data and the tools to analyse it. The experience of organisations and health systems that have adopted this approach is that collaboration^8^ is an essential ingredient for a learning community to be effective in improving care.^9^

From our seminar participants and previous research,^10^ ^11^ we identified a number of examples of LHS that have emerged in the United Kingdom. A strong theme from our seminar was that current developments in the NHS could enable LHS and that clinical teams, organisations, and health systems could do much more right now to extend the use of data for learning and improvement. However, the LHS examples we found are more focused on the data stage, and less on translating knowledge into action: it is hard to find examples where there is a continuous cycle of improvement driven by information, and where implementing improvements is part of usual ways of working.

## 3 METHODS

A seminar on LHS was held in January 2019, bringing together NHS leaders, academics, practitioners, and policymakers to share experience of LHS in the United Kingdom. The participants discussed what has been learned from developments in learning health systems to date, and what the lessons are for policy makers in the United Kingdom. The aim of the seminar was to develop policy lessons, considering what could be done at a national level to provide the right support for LHS locally, as well as what local systems could do to develop LHS.

There were 20 participants including clinical and informatics academics, chief clinical information officers, policy makers, data analysts, and quality improvement specialists. Participants worked in a range of organisations and settings including central bodies (NHS England and NHS Digital); acute hospitals, primary care, mental health services, and organisations working regionally to support the NHS (academic health science networks and commissioning support units). Participants were identified in order to represent a broad range of perspectives about LHS and were not funded to attend, except through their existing professional roles. Ethical approval for the seminar was not sought, but participants were notified that the findings from the seminar would be recorded and used to develop a report on policy implications of LHS.

Professor Charles Friedman, University of Michigan, shared insights from LHS in the United States, and Professor Jeremy Wyatt, Wessex Institute, discussed the tensions between innovation and evidence in a UK context.

Participants discussed the following questions:

- What are the quality improvement benefits to the NHS from developing learning systems?
- What skills, resources, and infrastructure would be needed to enable NHS organisations to be true learning systems?
- What are the implications of this for national policy bodies in supporting the development of learning systems, and evaluation and regulatory approaches?

Although challenges relating to interoperability and technical solutions, as well as consent, privacy and information governance are clearly very important, given limited time, these were not addressed at the meeting.

The discussion points from the seminar were grouped into themes.

In order to put the seminar discussion in context, additional evidence was obtained from a brief literature review on learning health systems. The aims of the literature review were to: identify whether there were areas of policy which were not covered by the seminar, to contextualise the themes identified from the seminar participants and to provide sources or examples relevant to the themes raised during the seminar discussion.

For the literature review, a Pub Med search was undertaken for: papers in English from the last 5 years; including the terms “Learning” and “Healthcare”/“Health”/“Health Care” and “System(s).” Titles and abstracts were screened to identify relevant papers: 177 papers were identified, of which 146 were found to be relevant to LHS policy. The majority of papers were descriptive: 42 papers described experience of developing a LHS, 31 presented methods used in LHS, and 30 provided an overview of the LHS approach. Additional references and examples were reviewed where these were mentioned by seminar participants, or from previous digital policy and research undertaken by the authors. Only references cited in the text are included in the reference list: a supplementary file containing the results of the literature search is available.

The research literature identified was reviewed alongside the themes which emerged from the seminar. The most significant theme from the research literature, which was not addressed in the seminar, was ethical issues relating to LHS.

Current UK policy initiatives relevant to LHS were summarised (Box 1‐3), and lessons for policy identified within each theme.

In developing policy lessons, we drew on our analysis of data from the seminar and evidence from the literature in addition to our own health policy expertise, for example, on innovation^12^ and that of expert reviewers. A draft briefing was reviewed by five of the seminar participants and a further four reviewers, with expertise similar to that of the seminar participants. Policy lessons have been grouped into those which can be addressed by national policy makers, and those which can be addressed locally by NHS organisations or health systems. The following can be related: national policymakers can address issues which create barriers or disincentives locally, and at the same time, local areas can sometimes take action themselves, within the constraints of national policy and guidance.

## 4 RESULTS

In the remainder of the paper, we examine the barriers for development of LHS and opportunities to overcome these. The barriers and lessons are grouped into the five themes, which emerged from synthesising the outcomes of the seminar and literature review.

### 4.1 Theme 1: research versus innovation and implementation

One cultural barrier highlighted by seminar participants is the higher status attributed to research than to innovation or implementation. Academic research is often competitive, hierarchical, and elitist, characteristics which are at odds with the collaborative open approach required for a learning health community. Clinicians are rewarded in career pathways for undertaking research, but there is not the same requirement or status associated with implementing improvements.

We heard from seminar participants that the experience from implementing learning communities is that clinicians engage because they feel they can make a difference to improving care. In the context of pressurised operational and clinical workloads in the NHS, clinicians can find it hard to spend time on improvement activities that are not valued by the system, particularly in the absence of incentives to do so.

This is a significant issue because there is a growing body of research^13^ on implementation science, which shows just how difficult implementing change can be. The institutions within the NHS are geared towards supporting research rather than innovation. The United Kingdom has a top down approach to implementing evidence; for example, evidence‐based guidance and approval for new treatments to receive NHS funding are controlled by the National Institute for Health and Care Excellence (NICE).^14^

#### 4.1.1 Balancing research with innovation: lessons for policy and the NHS

There is a need to increase the value of innovation in clinical careers, driving long‐term culture change. Potential actions we recommend could be considered include the following:

- Reviewing career “gateways” to ensure that innovation and improvement projects are given equal value to research. This could build on current revalidation requirements for clinicians to participate in clinical audit and improvement activities.
- Giving greater focus to improvement and innovation in clinical excellence/merit awards.
- Expand national programmes paying for clinical time to work on practical uptake, to match those for research.

### 4.2 Theme 2: analytical capacity and capability in the workforce

Seminar participants and reviewers emphasised that a shift to an LHS in which clinicians are routinely using data for improvement requires significant change to how NHS information and analysis functions are currently organised. The current reality is that analytics teams are usually managed as corporate functions, with a primary focus on analysis for regulation and performance rather than quality improvement and transformation.^15^

There are cultural barriers^16^ between clinical and informatics specialists. NHS IT and analytical staff are part of the clerical and administrative workforce rather than members of scientific grades such as lab technicians, limiting their training opportunities, salaries, and collaborative opportunities. This could reinforce a view in the NHS that analytical and informatics work is low status and belongs in the “back office” rather than being critical for transformation and quality improvement.^17^

In combination, these factors have contributed to a lack of specialist analytical capacity in the NHS.^15^ So while analytics methods^18^ already used for research and evaluation of health care could be applied in LHS, there is scarce capacity to do this in either the NHS or research communities. For LHS, this is a significant challenge, as methods used for robust analysis of large health datasets are not routinely undertaken in the NHS.

There is also a broader challenge to develop and strengthen digital skills in the NHS,^19^ including developing informatics skills among clinicians and managers, as well as quality improvement and clinical understanding among analysts. The Building the Digital Ready Workforce programme (see below) recognises and is working to address this challenge.

A major barrier is the current pay structure in the NHS that does not value technical expertise. Further, it means the NHS finds it difficult to compete with other sectors or commercial analytics organisations.^4^ Seminar participants noted that a number of significant national NHS analytics developments are outsourced, contributing to a cycle whereby skills and capacity in the NHS remain underdeveloped relative to third party organisations. A further impact of outsourcing is lack of transparency of methods—local organisations can find it difficult to replicate methods used in national tools, reducing opportunities for learning and causing duplication of effort.

Seminar participants noted that a huge amount of analytical effort in the NHS is duplicated, with multiple organisations producing routine reporting of the same performance measures. The current organisation of analytics support means that there is often competition between analytics teams rather than collaboration, arising from the NHS market for analytics skills.^20^ Some areas are tackling this by bringing together capacity across commissioners and providers, where local relationships allow this and a number of analytics hubs are developing within the NHS family, for example, in Academic Health Science Networks,^21^ commissioning support units,^22^ and shared informatics services.^23^ Hubs could provide the scale to develop and maintain specialist skills, alongside access to integrated datasets. Professional networks for NHS analysts, such as APHA (see below), are becoming established to address current barriers and provide a stronger voice for NHS analysts as well as training and development opportunities.

#### 4.2.1 Developing analytical capacity: lessons for policy and the NHS

National bodies should demonstrate leadership for analytics in the NHS in the way they commission and use analysis. This could include:

- promoting collaboration, learning, and transparency by ensuring the methodology and tools developed centrally are published and are accessible;
- encouraging development of analytics capability in the NHS through ensuring there is effective professional leadership for analytics at senior levels in national NHS organisations, for example, though appointing a chief analyst role and through regional analytics roles supporting local service transformation;
- addressing the growing challenge of pay and reward for analytical and informatics roles, which are currently relatively undervalued within the NHS framework.

Local NHS organisations and partnerships can also support the development of analytical capability. Actions could include:

- making openness and transparency in analytical methods the default approach to promote shared learning and improve consistency between organisations;
- consider the workforce implications and how local shared records (see LHCRs below) can develop the capability to make use of linked data;
- set an expectation for continuous learning, engaging in professional networking organisations, and collaboration within analysts' job descriptions and providing time for this in jobs. This could include encouraging the use of MOOCs, wikis, and other knowledge‐sharing tools

### 4.3 Theme 3: ethical issues and LHS

The research literature indicates that a further set of ethical concerns^29^ have emerged as LHS have been implemented in the United States about how patients are involved in situations where evidence is gathered at the point of usual care, rather than in a research setting. On the one hand, if it is not clear which treatment is best, clinicians have a duty to monitor the impact of different treatments. This also has a benefit from widening the populations for which evidence is available, as most clinical trials are undertaken for a narrow group of patients. On the other hand, there are concerns^30^ about patient's consent for different treatment options: how patient's data is used for purposes beyond their usual care, ensuring that there are mechanisms for patients and communities to be involved, and transparency in the methods and findings from analysis.

Pragmatic RCTs^31^ provide one route to widen participation in clinical trials, but the LHS can take this much further through routinely randomising treatment at the point of care^32^, where evidence about the best treatment is lacking (referred to as clinical equipoise). When there is genuine equipoise between two commonly used treatments, not doing a point of care trial to find out which has a better risk/benefit profile seems unethical. Pragmatic RCTs could enable clinically important questions that can be answered fast based on studies carried out in routine care settings on a full range of participants.

Research governance frameworks have been developed to address these issues in a research context: current guidance from the Health Research Authority (HRA), the body responsible for research governance in the United Kingdom, distinguishes between service evaluation and research. However, the guidance may be difficult to apply in the same way for research carried out at the point of care.

#### 4.3.1 Addressing ethical issues: lessons for policy and the NHS

Research bodies need to respond to the growing opportunities for research using large routine datasets. Potential actions could include:

- HRA could re‐examine guidance on studies carried out at the point of care and other novel approaches, considering the balance between population benefits, where evidence is currently limited and the risks to the individual patients recruited
- The National Institute for Health Research could launch a themed call or new programme with accelerated decisions, designed to fund point of care trials to investigate NICE‐identified research recommendations, using routine data.

### 4.4 Theme 4: robust data access and governance arrangements

The digital health data that an LHS would use is generated on multiple systems. A single hospital trust will have multiple, often dozens, of different clinical systems collecting data for specific purposes. Bringing this data together requires physical infrastructure for storing and processing data, as well as mechanisms for linking data, which may not share common patient identifiers. Providing access to such linked data to analysts based across multiple organisations, or combining with patient‐generated data^33^, adds further layers of complexity.

There are different ways in which data linkage and access can be achieved, and importantly for the development of LHS, the solution used is interdependent with arrangements for data access and governance. For example, some solutions “pull” selected data from different systems at the time it is needed for clinical care or analysis, while in others, data is routinely “pushed” into a common data warehouse for storage. The push model generally requires stronger data governance arrangements, because purposes of data access are less specific and requires greater public trust, but is also necessary for analytical work at scale.

General Data Protection Regulation (GDPR)^34^ clarifies that implied consent may be used for sharing confidential patient information for an individual's care. Where an individual's care team is involved in local clinical audit, data can also be shared with implied consent. Confidential patient information can also be shared without consent if doing so is considered to be in the public interest or where there is another mandatory legal requirement for the data, such as a court order.

Linked but anonymous data can be shared under GDPR. However, in the context of an LHS that is aiming to link multiple sources of patient level data, the task of linking data across multiple systems is challenging if these are held by different organisations. A lack of clarity about how to meet information governance requirements, while also providing various stakeholders with access to linked data, has been a significant barrier to evaluating the impact of new models of care.^35^ Further, effectively pseudonymising patient data becomes more challenging because there are more data points about a person. This could make them uniquely recognisable, even with no identifiers. Although there are technical solutions to reduce risks of unintended disclosure, as part of privacy enhancing technologies, they are not in widespread use in the NHS. Patients and the public now have the opportunity to opt‐out of their confidential (but not anonymous or depersonalised) data being used for research and planning purposes, but a large proportion of people opting out will reduce the value of analysis undertaken using the data.^36^

The NHS has struggled to develop governance arrangements which enable information to be accessed for the benefit of individual patient care and research, while also meeting regulatory requirements and patient expectations: further guidance is expected shortly to address this.^37^ The vast majority of people in the United Kingdom trust the NHS and most are happy with it using data, even for secondary uses.^38^ However, some people have concerns^39^ about who accesses their health data. Missteps in the development of shared records have led to a lack of public trust^40^ in data sharing, and have also contributed to a high level of risk aversion among clinicians and data custodians.

These challenges are recognised within new initiatives to extend the use of data and analytical methods in health research (see below). Although these programmes differ in their focus and scope, they all include work to develop the governance and public engagement framework for managing and using data beyond direct patient care, as well as the data and technical capability required. These frameworks are starting to empower patients and the public to take greater control of their data, and also need to involve them in how their data will be used and analysed.

#### 4.4.1 Enabling data access and robust governance arrangements: lessons for policy and the NHS

- The considerable investment through HDR UK, Digital Innovation Hubs and LHCRs is welcome and should have long‐term impact. However, there is a need to build quickly on what can be done using existing infrastructure, and the key to this is likely to be promoting the LHS ethos of collaboration and iterative development.
- Infrastructure programmes need to consider the current NHS environment and the need for clinical ownership of data access and analytics; it is not clear that the current models for implementation of LHCRs, for example, will develop this.
- Locally, the strongest message from health systems that have successful linked data is that clinical ownership is key to this, along with active programmes to develop public trust.^4^

### 4.5 Theme 5: high quality data

EHRs and the digital capture of data on observations, tests, treatment, and outcomes, will lead to an exponential increase in the amount of data potentially available for analysis. However, seminar participants emphasised that the existence of this data won't automatically translate into useful information and learning unless the data is of sufficiently high quality and in a form that can be readily analysed.

The NHS has a long history of collecting data routinely and using this for research, for example, Hospital Episode Statistics^46^—a structured dataset covering hospital activity. Even with this structured data, there are challenges to data quality^47^ and completeness, such that interpreting variations between organisations or trends over time demands careful scrutiny by experienced analysts of potential coding differences.

Primary care services in the United Kingdom have used EHRs^48^ for many years, but some of this data is often unstructured free text, there is limited consistency between clinicians in how data is recorded (except in data required for performance payments), practices have limited analytical capacity, and there has not been a widespread culture of sharing data between practices for learning. While there are analytical solutions^49^ to these problems, we need to recognise that more data will not automatically translate into learning without incentives or investment to achieve this.

That having been said, the NHS already has a considerable programme of work to develop data standards (see box).

Maintaining and developing standards is a continuous process and there are ways to make the most of the existing data. Where there is variation in data standards in use, mapping between different sources can enable data to be analysed, without there being a single data source or standards. For example, this approach has been successfully applied across primary care sources^50^ in different countries.

In addition, there are important gaps in data collected about patient outcomes. Patient Reported Outcome Measures (PROMs) are widely used in research but are not part of most routine care. There are exceptions such as outcomes for psychological therapy^51^ and joint replacement^52^. There is growing interest in using PROMs at the individual patient level,^53^ as part of patient care. This can support shared decision making, inform clinical decisions, and enable care to be tailored to individual needs. Seminar participants emphasised that routine use and collection of PROMs could play a critical part in enabling services to monitor outcomes, and drive improvements in care.

#### 4.5.1 Ensuring high quality data: lessons for policy and the NHS

To make the most of existing data, and enable rapid use of new sources of digital data, the NHS needs to take a strategic approach to developing data. This includes:

- promoting ongoing work on data structures and definitions and provide clear guidance for local organisations on interpreting and delivering the standard;
- building standards into the development of new data collections, for example, through the procurement process;
- addressing the current gap in routine collection of outcomes data and specifically the dearth of patient‐reported outcome measures—outcomes data is critical in completing the learning cycle and in enabling comparisons of quality of care;
- building analytics requirements into local digital plans so getting information out of EHRs and into a form which can be used for improving care is part of the plan from day one and is not seen as an optional extra to be addressed in the future.

## 5 DISCUSSION

LHS aim to address the challenge of ensuring that the huge growth in digital health and care data can be used to improve care, to shorten the timeframe of improvement projects and ensure these are based on real‐world data. In the United Kingdom, as elsewhere, policymakers are depending on digital innovation, driven by better use of data about current health service performance to enable service transformation and a more sustainable health system.

In this paper, we have identified a number of barriers to achieving these aims, which need to be given greater consideration in order to realise the benefits of a digital NHS. While there are excellent examples of UK organisations and networks that are moving on this journey already, we have also identified ways in which national and local organisations could promote and accelerate the development of LHS.

In addition to the cultural factors that provide motivation and enable learning, several building blocks contribute to effective LHS that can operate at scale. The NHS is already investing in some of these building blocks, but there are some gaps and significant challenges to achieving current aspirations.

A key question for the UK NHS, and likely other health systems, is whether the right incentives are in place to enable better use of health data for improvement at scale, both in terms of driving the right culture and developing the infrastructure. Currently, there are too few external incentives for clinicians to engage in quality improvement—given the absence of outcomes measurement and the lower status of implementation compared with original research. And at the moment, NHS analytics functions are more focused on regulation and performance than they are on quality improvement and transformation.

An additional challenge, both locally and nationally, is that investment to develop the infrastructure needs to be made by different groups or organisations from the learning health communities who would benefit. This mirrors the experience of local shared record programmes, which required individual NHS organisations to invest financially and collaborate, for the benefit of system wide infrastructure. While organisations are likely to benefit in time, the experience in the United Kingdom and elsewhere is that it can take a number of years for this to happen.

This paper focuses on the UK policy context. However, many of the same challenges have been experienced in other countries. The LHS concept should prompt the NHS to look more closely at how we can improve the use of data and promote collaboration rather than competition. There is scope for national policy and interventions to promote the cultural shifts that will be needed for more data to translate into improved care.

### 5.1 Limitations and further research

This paper is based on a seminar with 20 participants and a brief review of existing evidence. Perspectives expressed during the seminar informed much of the narrative and differing perspectives will of course exist, which are not captured here. While every effort was made to triangulate findings with existing literature, building learning health systems in an NHS context is a relatively new area and little literature exists in this area. Rather, the majority of literature is focused on learning health systems more broadly and how they have developed elsewhere. What's more, the work drew on a brief literature review and therefore key studies may be missing. Nevertheless, this paper provides a starting point for others to start conceptualising learning health systems in an NHS context and illuminates where further thought and action is needed.

## CONFLICT OF INTEREST

None declared.
